# Editorial: 2021, A New Chapter

**DOI:** 10.5334/cpsy.62

**Published:** 2021-04-21

**Authors:** Rick A. Adams, Xiaosi Gu

**Affiliations:** 1 Centre for Medical Image Computing, Dept of Computer Science, University College London, 90 High Holborn, London, WC1V 6LJ, UK; 2 Max Planck Centre for Computational Psychiatry and Ageing Research, University College London, Russell Square House, 10-12 Russell Square, London, WC1B 5EH, UK; 3 Center for Computational Psychiatry, Department of Psychiatry, Department of Neuroscience, Icahn School of Medicine at Mount Sinai, 1 Gustave L. Levy Place New York, NY 10029, US

We are very excited to announce a new chapter in the life of the journal Computational Psychiatry (CPsy). Starting from January 2021, we are the new Editors-in-Chief of CPsy, and the journal is being transferred from MIT Press to an open-access publisher, Ubiquity Press. We have taken this opportunity to make some exciting changes to the journal: we have reduced the article submission fees from $2500 to $2000 (and even less for short formats), we will now accept registered reports, and we will publish the reviews and authors’ responses of accepted papers. We strongly support diversity and inclusion in academia, and so we will do our best to accommodate authors who are unable to afford publishing charges – any such authors should contact us to discuss this.

We would like to pay tribute to our founding Co-Editors-in-Chief, Prof Read Montague and Prof Peter Dayan, who have passed the reins of the journal on to us. Their contribution to the journal and to the field as a whole has been immense, and continues to be so. We will be careful to maintain their high standards for the journal. We are also extremely grateful to our first Managing Editor, Cassie Carrin, who worked well beyond her allotted hours to ensure the journal ran smoothly and efficiently; and we look forward to working with our new Managing Editor Matt Heflin.

Our subject-matter of interest remains unchanged: we will still cover applications of computational methods to analysing behaviour or biological measures such as neuroimaging data – or the relation between the two – in mental health or mental disorder. Computational Psychiatry is quickly becoming established as a field (Huys et al., 2021): since this journal was founded in 2016, the field has been covered by some excellent textbooks (Anticevic & Murray, 2017; Series, 2020), teaching courses have become established in London, Zurich, New York, and at satellite meetings of major conferences, and the Transcontinental Computational Psychiatry Workgroup seminars are thriving (see www.cmod4mh.com for more). Standards for the design and analysis of computational modelling experiments have become accepted (Palminteri et al., 2017; Wilson & Collins, 2019), as have those for sharing both models and data (Poldrack et al., 2019).

The most important future challenge for Computational Psychiatry – already much discussed by many in the field (Browning et al., 2020; Redish et al., 2017) – is to translate the knowledge gained by the field into *tangible* clinical benefits. One of the key first steps in this challenge is to improve the within-subject reliability of computational measures; related to this is the need to focus more on longitudinal within-subject measures rather than cross-sectional ones (Huys et al., 2021). We will especially welcome submissions that meet these and related challenges.
